# Rare Localized Adverse Reaction to Botulinum Toxin Type A in a Patient Without Allergic History: A Case Report

**DOI:** 10.1093/asjof/ojag012

**Published:** 2026-01-20

**Authors:** Roselaine Roratto Muner, Amanda Ambrosio Moreira, Isabela Espasandin, Andrea Damas Tedesco, Alan Cristian Marinho Ferreira, Antony de Paula Barbosa

## Abstract

Botulinum toxin type A is widely used in aesthetic and therapeutic medicine and is considered safe. Nonetheless, rare adverse reactions can occur, even in patients with no previous allergic history. The authors report the case of a 50-year-old woman who developed a localized inflammatory reaction characterized by erythema, edema, vesicles, burning, and pruritus following injections in the upper face. A reactivity test with another formulation in the subcutaneous tissue of the arm produced an even more severe response, suggesting greater immune reactivity in subcutaneous tissue compared with muscle. Considering the known differences among commercial formulations, including accessory proteins and stabilizers, the test excluded the hypothesis of an allergy specific to a single brand. Standard therapies with corticosteroids and antibiotics failed to produce improvement, whereas ozone therapy led to significant recovery. This case emphasizes the importance of individualized follow-up and highlights the need for further studies to elucidate the immunopathological mechanisms underlying such rare hypersensitivity reactions.

**Level of Evidence**: 5 (Therapeutic) 
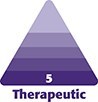

Botulinum toxin type A (BoNT-A) is one of the most widely used therapeutic and aesthetic agents worldwide, with established efficacy in reducing muscle contraction through inhibition of acetylcholine release at the neuromuscular junction.^1^ Its favorable safety profile has been documented in millions of procedures annually.^2^ Most adverse effects are mild and transient, typically presenting as localized pain, edema, or erythema at the injection site. Severe adverse reactions, such as allergic or pseudo-allergic responses, are exceptionally rare and insufficiently characterized in the literature.^3^

BoNT-A is available in several commercial formulations, which differ in their manufacturing processes, excipients, and protein complexes. These differences can impact both clinical efficacy and immunogenic potential.^4-7^ We describe here a rare case of a hypersensitivity reaction in a patient with no allergic history following Nabota (Daewoong Pharmaceutical Co., Ltd, Seoul, South Korea) injection, which was further investigated with a reactivity test using Dysport (Ipsen/Galderma, Uppsala, Sweden). The more severe response observed in subcutaneous tissue compared with intramuscular injection underscores the importance of understanding tissue-specific immune environments and the potential immunological role of formulation components.

## CASE PRESENTATION

In 2021, a 50-year-old woman presented seeking aesthetic treatment for dynamic lines in the upper third of the face. She had no personal or family history of allergies, no chronic illnesses, and no regular medication use. Her only previous aesthetic intervention was medium-depth chemical peeling. She reported no systemic hypersensitivity to insect bites or contact allergens and had undergone multiple invasive procedures without adverse reactions to needles or other injection materials.

**Figure 1.**
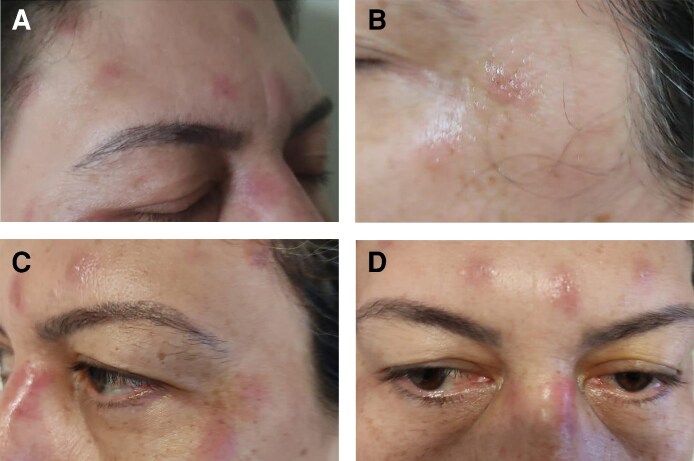
(A-D) Erythematous and edematous lesions with vesicles in the region of the frontal and orbicularis oris muscles presented by a 50-year-old woman, 12 h after application of PrabotulinumtoxinA (Nabota) to the area.

**Figure 2.**
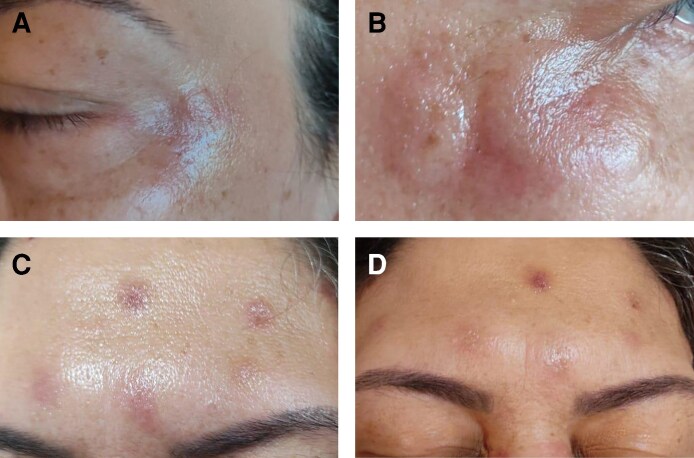
Persistent vesiculobullous lesions in the region of the frontal and orbicularis oris muscles, associated with itching and burning sensation, presented by a 50-year-old woman. (A-D) Records of the reaction at 1, 2, 4, and 70 days after application, respectively, after application of PrabotulinumtoxinA (Nabota) to the area.

Treatment was performed with PrabotulinumtoxinA (Nabota, Daewoong Pharmaceutical Co., Ltd, Seoul, South Korea), reconstituted with 100 units in 2 mL of sterile 0.9% saline solution. A total of 34 units were injected into the glabellar complex, frontalis, orbicularis oculi, and nasalis muscles. Within 12 h, she developed localized pain, erythema, swelling, pruritus, burning, and vesicular lesions in the frontal region (Figure 1). Figure 2A-C shows records of the reaction on the first, second, and fourth day after application, respectively. Figure 2D shows a record of the same region 70 days after application. These episodes recurred intermittently for up to 2 years.

**Figure 3.**
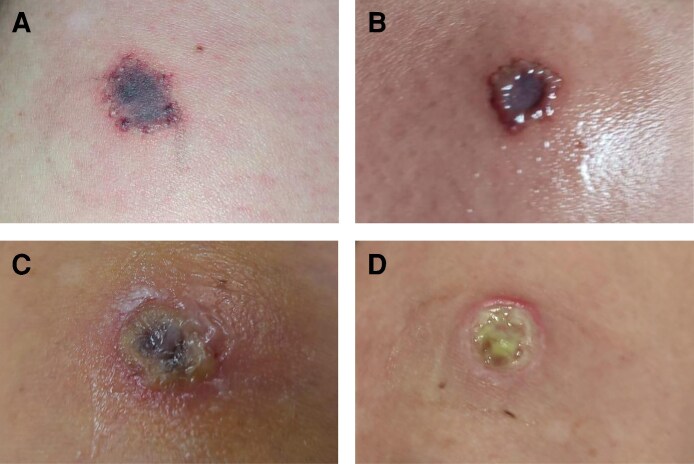
Severe erythema, edema, vesiculation, and ulceration presented by a 50-year-old woman at the site of subcutaneous application of PrabotulinumtoxinA (Dysport) in the arm. (A-D) Records of the reaction at intervals of 1, 4, 7, and 10 days after application to the arm, respectively.

To assess whether the reaction was formulation specific, a reactivity test was performed 4 days after the initial application with AbobotulinumtoxinA (Dysport, Ipsen/Galderma, Uppsala, Sweden), injected subcutaneously into the arm at a dose of 1 unit. The test triggered intense erythema, edema, vesicles, ulceration, and pain, exceeding the severity of the facial reaction. Figure 3A-D shows records of the reaction at intervals of 1, 4, 7, and 10 days after application to the arm, respectively. This result suggested heightened immune reactivity in subcutaneous tissue, attributed to its richer population of mast cells, dendritic cells, fibroblasts, and vascular supply.^8,9^ No systemic manifestations occurred.

Five days after the initial application, the patient was instructed by the dermatologist and began treatment with oral prednisone (20 mg every 8 h, tapered) and amoxicillin (500 mg every 8 h for 7 days) produced no clinical improvement. Laboratory investigations, such as eosinophil count or autoimmune screening, were not performed at the moment.

**Figure 4.**
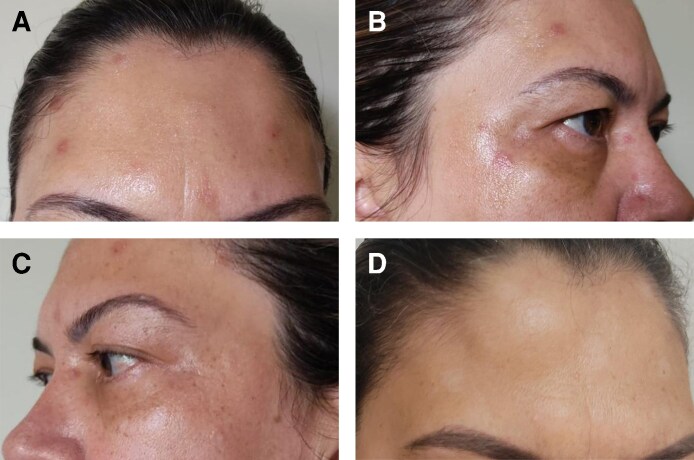
Postozone therapy: significant resolution of inflammatory lesions with residual hypochromia in the frontal region presented by a 50-year-old woman. (A-C) Shows the 5-day records and (D) shows the 1-year and 3-month records after the initial application of PrabotulinumtoxinA (Nabota) to the area, respectively.

**Figure 5.**
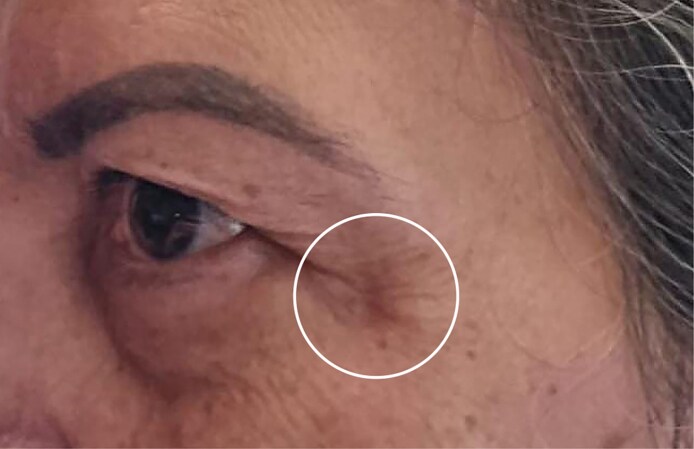
After 2 years of follow-up, the 50-year-old woman still presented with occasional vesicular flare-ups, which were milder and did not present with systemic manifestations.

Because of the persistence of symptoms, 7 days after the initial application, an ozone therapy protocol was initiated in the facial and arm regions where Nabota and Dysport botulinum toxin applications were performed, respectively. In both regions, a positive clinical response was observed from the first application, which represented an important milestone in the therapeutic process, considering that no improvement had been obtained with other approaches until then. On the face, the protocol consisted of daily topical application of ozonized oil every 4 h to the affected area and local injections twice a week of 5 μg of ozone, with 0.5 mL per point, distributed in the region where small papules and blisters had appeared shortly after the application of botulinum toxin. At these points, the application of ozone has promoted an immediate reduction in the volume of the papules and blisters, as well as inflammation, although there was a partial recurrence of symptoms, justifying the maintenance of serial local applications. In addition, rectal ozone was also applied at 120 mL with 5 μg daily for 8 days, followed by weekly sessions. In the arm region, where a subcutaneous lesion had formed, the same therapeutic protocol was adopted, applying ozonized oil topically and injecting ozone around the lesion, continuing treatment until complete tissue healing. After 30 days of continuous treatment, significant clinical improvement was observed in both regions, with resolution of erythema and vesicles and only slight residual hypopigmentation. A significant reduction in the inflammatory process, evident tissue regeneration, and total resolution of the skin lesion on the arm was also observed. Even with progressive improvement of blisters, edema, and erythema, it was observed that, at times, the skin manifestations reappeared intermittently. In these episodes, new ozone applications were performed on the same affected areas, resulting in almost immediate remission of inflammatory signs, which reinforces the anti-inflammatory and modulating action of ozone on the local tissue. Figure 4A-C shows the 5-day records, and Figure 4D shows the 1-year and 3-month records of the application of PrabotulinumtoxinA (Nabota) to the area, respectively. At 2-year follow-up, the patient continued to report sporadic vesicular episodes, although less intense and without systemic involvement (Figure 5).

## DISCUSSION

This case illustrates an uncommon, localized hypersensitivity reaction to BoNT-A in a patient without an allergic history. Contamination of the vial or injection materials was excluded because the same batch was used in another patient without adverse effects. Infectious causes were also considered unlikely, given the absence of systemic manifestations and the poor response to the antibiotic. Allergic contact dermatitis to the needle was ruled out since the patient had previously undergone multiple procedures with identical needles without complications.

A critical element was the reactivity test with Dysport. Both Nabota and Dysport contain the 150 kDa botulinum neurotoxin but differ in molecular complex size, excipients, and accessory proteins. Nabota is supplied as a 900 kDa neurotoxin complex stabilized with 0.5 mg of human serum albumin and 0.9 mg of NaCl in a 100 U vial, whereas Dysport has a total molecular weight of ∼300 to 900 kDa, consisting of the 150 kDa neurotoxin associated with hemagglutinin and nonhemagglutinin accessory proteins, and is stabilized with 2.5 mg of lactose and 125 µg of albumin in a 500 sU vial).^6,10^ Despite these differences, both toxins present a similar amount of active neurotoxin—Nabota contains ∼0.75 ng/100 U and Dysport about 1.35 ng/250 sU.^4-7,11,12^

These proteins enhance stability but may also increase immunogenic potential. The more severe reaction observed after Dysport injection excluded the hypothesis of a formulation-specific allergy to Nabota and instead supports hypersensitivity either to the core neurotoxin or to accessory proteins shared among formulations. Furthermore, the greater intensity of subcutaneous reactions compared with intramuscular reactions reinforces the role of tissue-specific immunology: subcutaneous tissue harbors abundant immune cells and superficial vascularization that facilitate the release of inflammatory mediators, whereas skeletal muscle contains fewer resident immune cells and is less prone to exaggerated immune activation. The greater intensity of subcutaneous reactions compared with intramuscular reactions reinforces the role of tissue-specific immunology: subcutaneous tissue harbors abundant immune cells and superficial vascularization that facilitate the release of inflammatory mediators, whereas skeletal muscle contains fewer resident immune cells and is less prone to exaggerated immune activation.^8,9^

It is also pertinent to consider the hypothesis that a probable previous activation of the adaptive immune response by the first application of Nabota in the facial region sensitized the patient's immune system, contributing to the more intense reactions after the application of Dysport. Regarding the timing of the reaction, the lesions appeared in about 12 h after injection, which is compatible with a delayed-type hypersensitivity pattern rather than an immediate immunoglobulin E–mediated response. Typically, delayed hypersensitivity reactions (Type IV) develop between 12 and 48 h after antigen exposure and are mediated by sensitized T lymphocytes and macrophages rather than mast cell degranulation. This mechanism aligns with the clinical presentation in our patient, characterized by vesicular and bullous lesions with a slow and persistent course, as well as worsening upon re-exposure. Type IV reactions are frequently associated with specific alleles of HLA genes, with activation of CD4^+^ and CD8^+^ T lymphocytes, which are essential cells in the adaptive immune response.^13,14^ This supports the hypothesis that the first application may have led to immunological sensitization, inducing the formation of antibodies or memory T cells against some component of the toxin. However, considering the rarity of such reactions and the lack of confirmatory immunologic testing, this remains a hypothesis rather than a definitive conclusion. Although the initial approach was not to perform complementary tests, it is considered that the execution of immunological tests and laboratory evaluations would have been relevant to elucidate the etiological picture. The absence of this investigation represents a diagnostic limitation, because it is not possible to retrospectively confirm the exact origin of the process.

The persistence of vesiculobullous lesions for 2 years also suggests possible chronic sensitization to BoNT-A or residual inflammatory processes, mechanisms consistent with drug hypersensitivity syndromes.^15^ Notably, ozone therapy achieved significant improvement where corticosteroids and antibiotics failed, corroborating evidence of its anti-inflammatory, immunomodulatory, and reparative effects.^16,17^ Although not a conventional management strategy, its success here indicates potential as adjunctive therapy in select cases.

Despite the clinical relevance of this case, the report presents important limitations because of the absence of complementary tests and laboratory data that could have strengthened the analysis and allowed for a more precise immunopathological correlation. A more in-depth and systematic study with the patient would be necessary to corroborate the findings, but this faces significant ethical barriers, since repeating or inducing new exposures to the toxin could pose a risk of worsening the clinical condition.

## CONCLUSIONS

This case highlights a rare but clinically significant hypersensitivity reaction to BoNT-A, occurring despite the absence of allergic history. The stronger response observed in subcutaneous compared with intramuscular tissue emphasizes the importance of considering tissue-specific immune environments in the evaluation of adverse events. The comparison between Nabota and Dysport, which differ in protein complexes and excipients, underscores the role of formulation in shaping immune responses. Although this represents an isolated and rare presentation, further studies are required to clarify the immunopathological mechanisms suggested by this case and to guide safer therapeutic strategies in aesthetic practice.
